# Differential regulation of insulin-like growth factor binding protein-1 and -2 by insulin in the baboon (*Papio anubis*) endometrium

**DOI:** 10.1186/1477-7827-6-6

**Published:** 2008-01-31

**Authors:** Steven D Fleming, Asgerally T Fazleabas, Stephen C Bell

**Affiliations:** 1 Department of Obstetrics and Gynaecology, University of Sydney, NSW 2145, Australia; 2 Department of Obstetrics and Gynecology (MC808), College of Medicine, University of Illinois at Chicago, Illinois 60612-9998, USA; 3 Department of Obstetrics and Gynaecology, University of Leicester, Leicestershire LE2 7LE, UK

## Abstract

**Background:**

The purpose of this study was to examine the effect of insulin on expression and synthesis of IGFBP-1 and IGFBP-2 in the baboon endometrium in vitro.

**Methods:**

Baboon endometrial explants collected from cycling, ovariectomized, steroid-treated, simulated-pregnant and pregnant animals were cultured for 48 h in the presence or absence of insulin, with or without estradiol, progesterone and hCG.

**Results:**

Insulin clearly inhibited IGFBP-1 production and mRNA expression in a time- and dose-dependent manner, whereas IGFBP-2 synthesis was not significantly affected. The inhibitory effects of insulin on IGFBP-1 were more evident in explants of non-pregnant tissue or tissue away from the implantation site. In the absence of insulin, synthesis of IGFBP-1 was induced in explants with low levels of de novo synthesis whereas IGFBP-2 synthesis was inhibited. This effect was potentiated by steroids and hCG in the explant cultures.

**Conclusion:**

Insulin differentially regulates endometrial IGFBP-1 and IGFBP-2 secretion in the baboon.

## Background

The insulin-like growth factor binding proteins (IGFBPs) include IGFBP-1 and IGFBP-2 that are non-glycosylated, low molecular weight IGFBPs that have a homologous amino acid sequence. They are important modulators of insulin-like growth factor (IGF) bioactivity. In this respect, IGFBP-1 and IGFBP-2 may potentiate the binding of IGFs to target cells via integrin receptors by virtue of the fact that both IGFBP-1 and IGFBP-2 have Arg-Gly-Asp (RGD) sequences at their C-terminus. The IGFs, IGF-1 and IGF-2, are mitogens that are involved in the regulation of endometrial cell proliferation, differentiation and apoptosis. Indeed, during the menstrual cycle the mitogenic effects of estrogen, balanced by the differentiating properties of progesterone, are mediated by the IGF system. In the primate, the major sites of synthesis of IGFBP-1 and IGFBP-2 are the liver and decidualized gestational endometrium, and the central nervous system, respectively.

During the menstrual cycle IGFBP-1 is a minor secretory product of the endometrial epithelium and stroma, however its production is markedly induced by progesterone during the late luteal phase [1-6]. Indeed, during pregnancy IGFBP-1 becomes the major soluble protein synthesised and secreted by the primate decidual cell [4,7-10]. Endometrial IGFBP-2 synthesis also increases during the luteal phase and pregnancy [6] and, similarly, appears to be induced by progesterone within human endometrial stromal cells [11,12].

Clinical studies have shown that plasma levels of IGFBP-1 are rapidly downregulated in response to insulin administration [13-15]. Furthermore, work on the rat has demonstrated a differential level of induction of hepatic IGFBP-1 and IGFBP-2 synthesis by insulin deficiency [16]. This *in vivo *regulation of IGFBP-1 and IGFBP-2 by insulin has been confirmed by *in vitro *studies in the rat [17] and human [11,18-20]. Studies of the liver IGFBP-1 proximal promoter region have revealed an insulin response sequence adjacent to the glucocorticoid response element, and analysis of these DNA sequences has demonstrated insulin inhibition and glucocorticoid or progesterone stimulation of IGFBP-1 transcription [21-25].

The earlier studies of the differential inhibitory effects of insulin on IGFBP-1 and IGFBP-2 gene expression [11,16] prompted us to examine this further in the primate, using the baboon as a model. The objective of this study, therefore, was to examine the effect of insulin on protein synthesis, secretion and mRNA steady state levels of IGFBP-1 and IGFBP-2 in the baboon endometrium *in vitro*.

## Methods

### Tissue samples

Baboon endometrial specimens were obtained from animals either on day 10 post-ovulation (n = 3), following ovariectomy (n = 3) and subsequent treatment with steroids (estradiol plus progesterone; [26]), between days 25 and 30 of pregnancy (n = 1) [27], or on days 24 or 25 post-ovulation (n = 2), following long-term treatment with steroids [28]. Steroids (Sigma-Aldrich, St. Louis, USA) were administered via 6 cm silastic implants using one of the following regimens: either 14 days of estradiol, 14 days of estradiol followed by 14 days of estradiol plus progesterone, or 14 days of estradiol followed by 14 days of progesterone [26]. The Animal Care Committee of the University of Illinois approved all of these experimental procedures.

### Explant culture

Tissue explants (n = 2) of endometrium from the different treatment groups of baboons were cultured in serum-free MEM devoid of insulin (Gibco/BRL, Gaithersburg, USA) over 48 h, controlling for differences in explant weight as described previously [29]. Explants were cultured in the presence or absence of 1.5 μM insulin (Sigma-Aldrich, St. Louis, USA). Some of the baboon explants were also cultured in the presence of the following hormones: estradiol (36 nM), progesterone (1 μM) and hCG (Profasi; EMD Serono, Rockland, USA; 0.5 IU/μl). The concentration of insulin in the complete medium was within physiological levels [30] whilst the concentrations of estradiol, progesterone and hCG were comparable to those assayed in conditioned media from the explant culture of day 25 pregnant baboon placentae [27]. Explant-conditioned media was harvested and dialysed against 2 mM Tris/HCl (pH 8.2) to remove the salt and amino acids that would otherwise interfere with gel electrophoresis and western blotting, and was replaced with a fresh basal medium at 20–24 h intervals over a short-term culture period of two days. Protein was quantified following dialysis using the Lowry method. Equal amounts of protein (50 μg) from the conditioned media were lyophylised and dissolved in Laemmli buffer for gel electrophoresis. At the termination of the culture period, the tissue was immediately rapidly frozen at -70°C for RNA or protein extraction.

### SDS-PAGE, western blotting and immunoassay

Conditioned media from the explant cultures was separated by one-dimensional gradient (5–17%) SDS-PAGE (20–200 μl/lane) under reducing conditions, employing molecular weight markers, as described previously [7]. Protein loading was checked using Ponceau stain, as described previously [29]. Gels were stained for 1 h, shaking at room temperature, with 0.1% (wt/vol) Coomassie brilliant blue in 50% (vol/vol) methanol and destained prior to western blotting.

Proteins were transferred from the gels to nitrocellulose membranes by electroblotting, as described previously [5]. Western blots of the conditioned media harvested from the explant cultures were first probed with a polyclonal antibody to IGFBP-2 (UBI, Lake Placid, USA) at a titre of 1:2000, the immunoreactivity being visualized using alkaline phosphatase. Using the same membranes, they were then probed with a monoclonal antibody (B_2_H_10_) to IGFBP-1 [10,31] at a titre of 1:2000, the immunoreactivity being visualized using an enhanced chemiluminescence kit (Amersham, Arlington Heights, USA). The results from some blots were semi-quantified using densitometry [6,32].

Immunoreactive IGFBP-1 in explant culture medium was assayed using a specific immunoradiometric assay kit (DSL, Webster, USA). Assays were performed in triplicate.

### Extraction of RNA and northern blotting

Total RNA was extracted from the tissue explants following short-term (two days) culture using TRI reagent (Molecular Research Center Inc., Cincinnati, USA). The concentration and purity of RNA was estimated using 268/28 nm ratios on a spectrophotometer. This RNA (20 μg) was then separated on a 1.2% agarose-formaldehyde gel and transferred to nitrocellulose. These northern blots were hybridised at high stringency with ^32^P-labelled cDNA probes (1.2 Kp and 795 bp, respectively) to human IGFBP-1 [10] and IGFBP-2 [33].

## Results

### Effects of insulin on IGFBP-1 and IGFBP-2 production

In the baboon explant cultures it was found that, generally, IGFBP-1 and IGFBP-2 production was progesterone-dependent. However, the synthesis of IGFBP-1 was inversely proportional to the level of insulin in the culture medium whereas that of IGFBP-2 was directly proportional to it.

**Figure 1:**
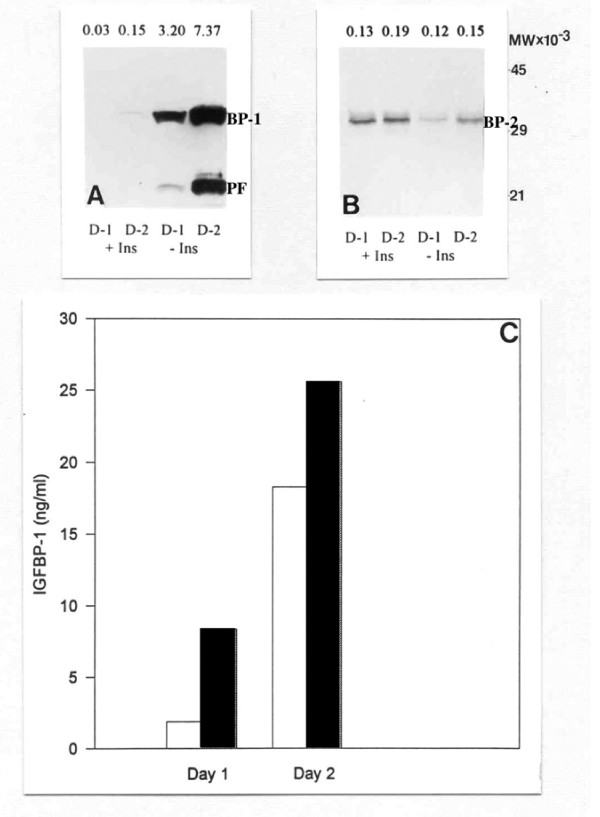
**Effect of insulin on IGFBP-1 and IGFBP-2 at day 10 post-ovulation**. The effect of insulin (+/- Ins) on IGFBP-1 (BP-1) and IGFBP-2 (BP-2) secretion in culture media taken from endometrial explants (n = 2) at day 10 post-ovulation. Media samples were analysed after 24 h (D-1) and 48 h (D-2) incubation by western blotting for IGFBP-1 (A) and IGFBP-2 (B), and by ELISA for IGFBP-1 (C). Values above each band in A and B represent their optical density. PF: Proteolytic fragment of IGFBP-1. Open and closed bars in C represent IGFBP-1 levels released by explants from two different animals in the absence of insulin.

Western blotting of conditioned medium harvested from explants of endometrium collected from two baboons at day 10 post-ovulation demonstrates the effect that the presence or absence of insulin in the culture medium has on the production of IGFBP-1 and IGFBP-2 (Figure 1). For IGFBP-1, two bands can be seen at around 32 kD and 19 kD, the latter representing a proteolytic fragment [6,32]. Levels of IGFBP-1 are virtually undetectable in the presence of insulin whereas they are clearly evident in the absence of insulin, levels freshly released on day two of culture being approximately double those on day one (Figure 1A). Assay of these media samples for immunoreactive IGFBP-1 confirms the western blotting data but does show that IGFBP-1 is produced in varying amounts by different explants in the absence of insulin (Figure 1C). In contrast, IGFBP-2 is detectable whether insulin is present or absent, levels being higher in the presence of insulin, but not changing markedly from day one to day two of culture (Figure 1B).

**Figure 2:**
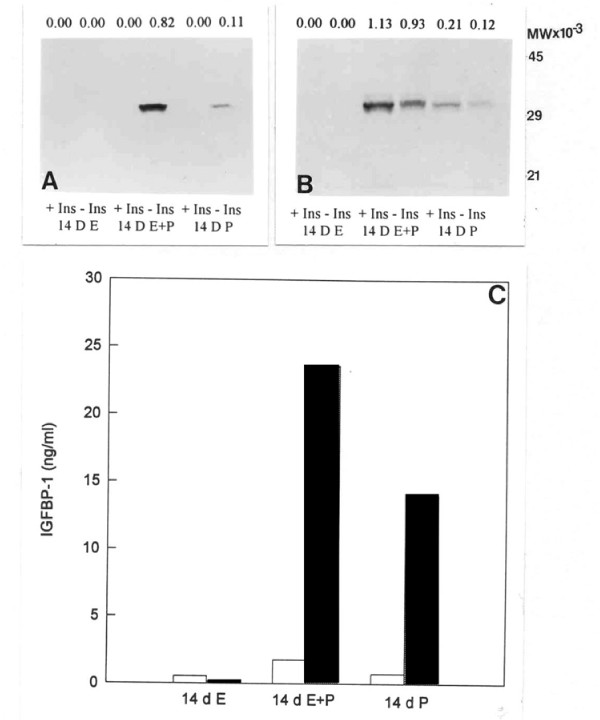
**Effect of insulin (+/- Ins) on IGFBP-1 and IGFBP-2 secretion in steroid-treated ovariectomized baboons**. Endometrial explants (n = 3) were obtained following steroid treatment and incubated in vitro. Media samples were analysed by western blotting for IGFBP-1 (A) and IGFBP-2 (B), and by ELISA for IGFBP-1 (C) after 24 h incubation. 14 d E: Treatment with estradiol for 14 days; 14 d E+P: Treatment with estradiol for 14 days followed by treatment with estradiol plus progesterone for 14 days; 14 d P: Treatment with estradiol for 14 days followed by treatment with progesterone alone for 14 days. These treatments represent the late follicular, mid secretory and late secretory stages of the cycle, respectively. Values above each band in A and B represent their optical density. Open bars: + Insulin; Closed bars: - Insulin.

Western blotting of conditioned medium harvested from explants of endometrium collected from steroid-treated ovariectomized baboons demonstrates that the production of both IGFBP-1 and IGFBP-2 is induced by progesterone, and augmented by estradiol (Figure 2). Again however, levels of IGFBP-1 are virtually undetectable in the presence of insulin whereas they are clearly evident in the absence of insulin (Figure 2A). Assay of these media samples for immunoreactive IGFBP-1 confirms the western blotting data and shows the synergistic effect that estradiol and progesterone have *in vivo*, particularly when amplified by the absence of insulin (Figure 2C). Again, levels of IGFBP-2 are higher in the presence of insulin (Figure 2B).

**Figure 3:**
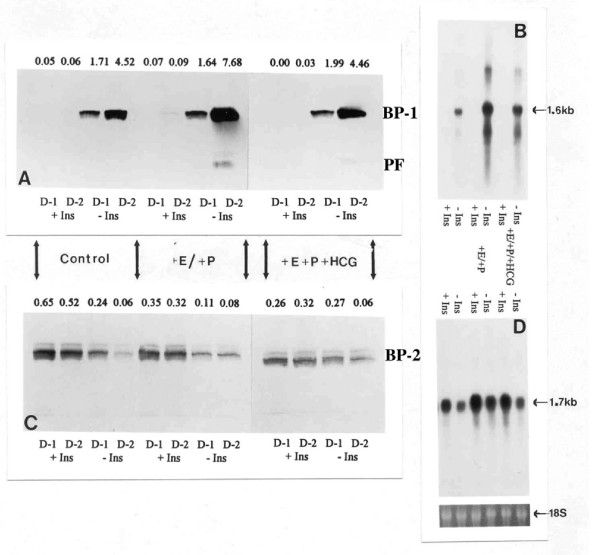
**Effect of insulin and hormones on IGFBP-1 and IGFBP-2 during simulated pregnancy**. The effect of insulin (+/- Ins) and hormones (hCG and/or estradiol and progesterone) on IGFBP-1 (BP-1) and IGFBP-2 (BP-2) synthesis in culture media taken from endometrial explants of intact baboons treated long-term (day 24–25) with steroids to simulate pregnancy (n = 2). Media and tissue samples were analysed by western and northern blotting for IGFBP-1 (A & B) and IGFBP-2 (C & D), respectively, after 24 h (D-1) and 48 h (D-2) incubation. Values above each band in A and B represent their optical density. PF: Proteolytic fragment of IGFBP-1; E: Estradiol; P: Progesterone.

Western blotting of conditioned medium harvested from explants of endometrium (collected from intact, long-term steroid-treated ovariectomized baboons) exposed to steroids and hCG in the culture medium demonstrates that the production of IGFBP-1 is enhanced, but only in the absence of insulin (Figure 3A). Again, higher levels of IGFBP-1 were observed on the second day of culture (Figure 3A), levels of IGFBP-2 being less variable, except in the absence of insulin where lower levels of IGFBP-2 were detectable on the second day of culture (Figure 3C). Furthermore, exposure to hCG appeared to inhibit steroid-induced synthesis and secretion of IGFBP-1 in the absence of insulin (Figures 3A and 3B).

**Figure 4:**
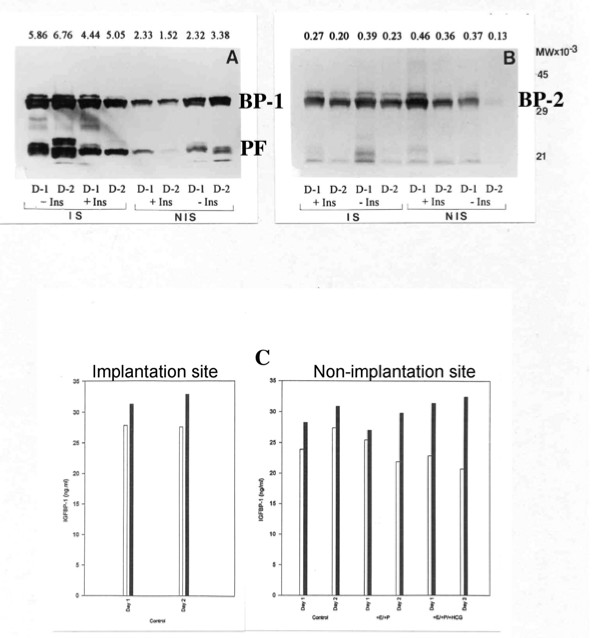
**Effect of insulin on IGFBP-1 and IGFBP-2 at the implantation and non-implantation sites**. The effect of insulin (+/- Ins) on IGFBP-1 (BP-1) and IGFBP-2 (BP-2) secretion in culture media taken from endometrial explants of the implantation and non-implantation sites of a day 32 pregnant baboon (n = 1). Media samples were analysed by western blotting for IGFBP-1 (A) and IGFBP-2 (B), and by ELISA for IGFBP-1 (C) after 24 h (D-1) and 48 h (D-2) incubation. Values above each band in A and B represent their optical density. PF: Proteolytic fragment of IGFBP-1. Open bars: + Insulin; Closed bars: - Insulin.

**Figure 5:**
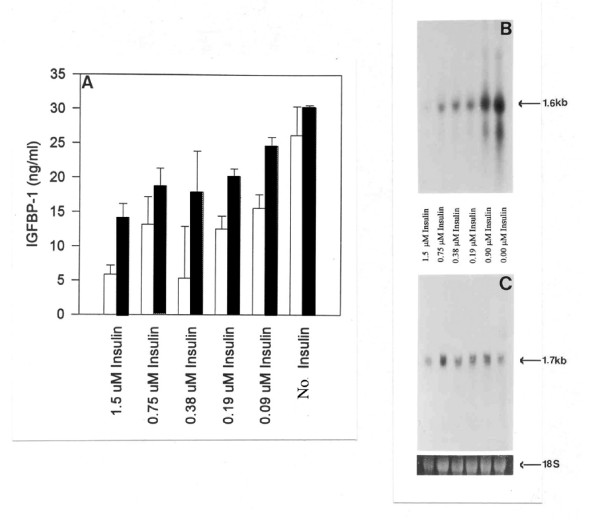
**Dose-response effect of insulin on IGFBP-1 in non-implantation endometrium at day 25 of pregnancy**. The dose-response effect of insulin on immunoreactive levels of IGFBP-1 in culture media taken from endometrial explants (n = 2) of the non-implantation site of a day 25 pregnant baboon. Media and tissue samples were analysed by ELISA for IGFBP-1 (A) after 24 h (Open bars) and 48 h (Closed bars) incubation, and by northern blotting, respectively, for IGFBP-1 (B) and IGFBP-2 (C).

Western blotting of conditioned medium harvested from explants of endometrium (either implantation site or non-implantation site tissue) collected from two baboons at day 32 of pregnancy, demonstrates the effect that the presence or absence of insulin in the culture medium has on the production of IGFBP-1 (Figure 4A) and IGFBP-2 (Figure 4B) is both modulated by pregnancy and is regionalized. In tissue from pregnant animals IGFBP-1 production is evident even in the presence of insulin, the inhibitory effect of insulin being less pronounced in tissue taken from the implantation site, where decidualization has begun (Figure 4A). Assay of these media samples for immunoreactive IGFBP-1 confirms the western blotting data (Figure 4C). Furthermore, a clear inverse dose-response relationship between the concentration of insulin in the culture medium and the production of IGFBP-1 from non-implantation site tissue can be seen (Figure 5), and this effect appears to be enhanced after two days of culture. Again, IGFBP-2 production is decreased in the absence of insulin, but only in tissue taken from the non-implantation site (Figure 4B).

### Effects of insulin on IGFBP-1 and IGFBP-2 expression

Northern blot analysis of endometrial RNA extracted from explant tissue (collected from a day 30 pregnant baboon) cultured with or without insulin and other hormones over a two-day period confirms that the production of IGFBP-1 and IGFBP-2 under these conditions is determined by their expression (Figures 3B &3D). Message for IGFBP-1 is only evident in the absence of insulin, the level of message being increased in tissue exposed to steroids and hCG (Figure 3B). Indeed, following a two-day culture of endometrial explant tissue a clear inverse dose-response relationship between the concentration of insulin in the culture medium and with the level of message for IGFBP-1 can be seen (Figure 5B). In contrast, message for IGFBP-2 is present both in the absence and in the presence of insulin, and the level of message appears to be increased in tissue that has been exposed to insulin (Figures 3D &5C).

## Discussion

To our knowledge, this is the first full report that characterizes the differential regulation of IGFBP-1 and IGFBP-2 by insulin in the baboon endometrium. We were able to demonstrate that physiological concentrations of insulin can inhibit IGFBP-1 production and mRNA expression in a time- and dose-dependent manner *in vitro*. As illustrated in Figure 5, insulin at a dose range from 0.09 μM to 1.5 μM resulted in decreasing levels of immunoreactive protein and mRNA for IGFBP-1. In contrast, the synthesis of IGFBP-2 was stimulated in the presence of insulin, although this increase was not statistically significant. Conversely, in the absence of insulin, we observed induction of the *in vitro *synthesis of IGFBP-1 in tissues exhibiting low levels of *de novo *synthesis. *Vice versa*, the synthesis of IGFBP-2 was inhibited in the absence of insulin.

Of particular interest is the relationship between the differentiation of endometrial stromal cells to decidual cells, whereby the endometrium of the menstrual cycle becomes the decidua of pregnancy, and the change in sensitivity of these tissues to insulin. Although insulin produced inhibitory effects on IGFBP-1 synthesis in mid-luteal and progesterone-treated baboon tissues, similar to those observed with explants from pregnant baboons, baseline secretory levels of IGFBP-1 were nevertheless lower than those seen in pregnancy. This demonstrates that the inhibitory effects of insulin on IGFBP-1 synthesis are evident prior to decidualization (when decidual cells begin their synthesis of IGFBP-1). Interestingly, in the baboon the inhibitory effects of insulin are more evident in non-pregnant tissue, or in pregnant tissue away from the implantation site. This suggests that when decidual synthesis of IGFBP-1 is initiated locally, during the establishment of pregnancy, the effects of insulin become less apparent. Hence, potential paracrine regulatory factors that influence IGFBP-1 synthesis may be induced and secreted during pregnancy.

IGFBP-1 is the major secretory product of the decidualized endometrium and *in situ *expression of IGFBP-1 mRNA is most abundant in those decidual cells adjacent to the invading extravillous trophoblast (EVT), which in contrast expresses IGF-II mRNA [34]. IGFBP-1 and IGFBP-2 possess an RGD sequence that can bind to RGD binding sites on cell surface integrins such as the integrin, α_5_β_1 _(also a receptor for fibronectin), and RGD-α_5_β_1 _binding is essential for the stimulation of trophoblast migration [35-38]. Indeed, it has been suggested that IGFBP-1 and IGF-II can stabilize each other at the EVT cell surface, potentially exerting a synergistic effect upon EVT cell migration and invasion [36,37]. Therefore, our observation in this study that the inhibitory effect of insulin upon IGFBP-1 secretion is diminished at the implantation site in the baboon endometrium may have physiological relevance.

Insulin could conceivably play an important role in the regulation of IGFBP-1 secretion, decidualization and implantation. In this respect, it has been found that insulin can inhibit IGFBP-1 secretion and gene expression in human endometrial stromal cells [11,39]. It has also been shown that cytotrophoblasts plated onto decidual multilayers are unable to penetrate the stromal cells unless decidual IGFBP-1 production is inhibited by insulin [40]. This impedence of trophoblast migration appears to be mediated by RGD-α_5_β_1 _binding since the permissive effect of insulin may be reversed with exogenous IGFBP-1 but not with IGFBP-3, which does not possess an RGD sequence [40]. Also, it appears that the actions of IGFBP-1 and IGF-II are further regulated by hCG [41-44]. Our observation of apparent inhibition of steroid-induced IGFBP-1 synthesis and secretion by hCG in the absence of insulin is consistent with this, and suggests a mechanism whereby embryo-derived hCG may conceivably augment implantation via increased receptivity and angiogenesis within the endometrium.

The corresponding changes in the levels of immunoreactivity and mRNA for IGFBP-1 and IGFBP-2 observed in this study indicate that regulation by insulin may occur at the level of transcription. Hence, changes in production of IGFBP-1 and IGFBP-2 in response to insulin presumably may reflect changes in the abundance of their mRNAs. Therefore, transcriptional or post-transcriptional regulation of IGFBP-1 and IGFBP-2 mRNA abundance may be crucial to the regulation of IGFs within the endometrium. In this respect, insulin may co-ordinate the bioactivity of IGFs with nutritional status through the regulation of IGFBPs. If so, such a mechanism could be important in the regulation of trophoblast invasion during implantation, perhaps particularly so in pathological states such as intra-uterine growth restriction. The inhibition of endometrial IGFBP-1 expression by insulin seen in this study correlates with other studies that have identified an insulin response sequence in the IGFBP-1 promoter, and similar phenomena have been observed in other *in vitro *systems [17-19,45]. Down-regulation of IGFBP-1 gene expression by insulin appears to be a phosphatidylinositol 3-kinase (PI3 kinase) dependent mechanism [46]. One potential pathway downstream of PI3 kinase that has been proposed to regulate IGFBP-1 mRNA levels involves activation of protein kinase B/Akt [47]. Through this pathway, Akt-dependent phosphorylation of two forkhead transcription factors (Foxo1 and Foxo3) has been shown to directly inhibit their binding to the insulin response element in the promoter of the IGFBP-1 gene via their nuclear exclusion [48-53]. An alternative pathway downstream of PI3 kinase, independent of Foxo1 and Foxo3 modulation, has also been proposed to regulate IGFBP-1 gene transcription in response to insulin [54,55]. This pathway involves the mammalian target of rapamycin, a protein with a catalytic domain homologous to that of PI3 kinase.

## Conclusion

In conclusion, to our knowledge this is the first full report that confirms in the baboon endometrium the central role that insulin has in the regulation of IGFBP synthesis, as reported by others in different experimental models. As such, this work further demonstrates the value of the baboon as an experimental model for the human in our understanding of the events surrounding decidualization, implantation and pregnancy.

## Authors' contributions

ATF and SCB were responsible for the conception, design, funding and supervision of this work and SDF was responsible for writing the manuscript. ATF and SCB read and approved the final manuscript.
